# A liaison between chaperone-mediated autophagy and exocytotic lysosomes controls the dendritic metastable proteome

**DOI:** 10.1080/15548627.2023.2274256

**Published:** 2023-10-24

**Authors:** Katarzyna M. Grochowska, Michael R. Kreutz

**Affiliations:** a Leibniz Group ‘‘Dendritic Organelles and Synaptic Function’’, Center for Molecular Neurobiology, ZMNH, University Medical Center Hamburg-Eppendorf, Hamburg, Germany; b Research Group Neuroplasticity, Leibniz Institute for Neurobiology, Magdeburg, Saxony-Anhalt, Germany; c Center for Behavioral Brain Sciences, Otto von Guericke University, Magdeburg, Saxony-Anhalt, Germany; d German Center for Neurodegenerative Diseases (DZNE), Magdeburg, Saxony-Anhalt, Germany

**Keywords:** CMA, DLG3/SAP102, exocytosis, GRIN/NMDAR, LAMP2, lysosomes

## Abstract

The neuronal metastable proteome includes several aggregation-prone proteins related to neurodegeneration. The complex morphology of neurons with very thin processes and enhanced protein turnover therefore necessitates efficient local machinery to remove excessive protein. In recent work we revealed that chaperone-mediated autophagy (CMA) provides cargo for dendritic exocytic lysosomes, a mechanism that serves in the rapid removal of disease-relevant, supersaturated proteins such as TARDBP/TDP-43 (TAR DNA binding protein) and HTT (huntingtin). We found that lysosomal exocytosis requires docking of the lysosomal protein LAMP2B to the glutamatergic receptor scaffold DLG3/SAP102 and that it is regulated by GRIN/NMDA (N-methyl-D-aspartate)-receptor activity. Thus, the small caliber of dendritic processes might impose a need for local disposal of aggregation-prone proteins like TARDBP and HTT. Moreover, we observed that lysosomal exocytosis might serve in both protein removal and modulation of synaptic processes, and the latter might be an inevitable consequence of the necessity for local disposal of CMA clients in dendrites.

## Article

Neurons are highly polarized cells with numerous thin processes that are decorated with several thousand synapses. The small caliber of axons and dendrites poses a threat and a challenge for the necessary dynamic changes in protein composition at synaptic sites that are associated with their plastic properties in learning and memory formation. Accordingly, synaptic proteostasis has been a hot topic in recent years but what has been overlooked is the question as to how activity-dependent removal of surplus proteins can be achieved. The failure of removal of aggregation-prone proteins is a hallmark in many age-associated neurodegenerative diseases and this failure results in interruption of protein transport, swellings, and varicosities in neurites but also impairment of synaptic function. Strikingly though, the majority of neuronal degradative machinery localizes to the somatic compartment, sometimes located several hundred micrometers from the synaptic site. Only very few studies reported the presence of lysosomes in dendrites, a convergence point of macroautophagy, chaperone-mediated autophagy (CMA), and endosomal pathways.

Initially, we asked the question how local lysosomes might control the dendritic proteome to maintain the function of distant synapses [1]. Our experiments in dissociated mature hippocampal neurons confirmed the presence of lysosomes in dendrites. Surprisingly, however, immunocytochemical and live-imaging experiments with dyes labeling active catabolic compartments revealed that the commonly and interchangeably used lysosomal markers, LAMP1 (lysosomal-associated membrane protein 1) and LAMP2 do not always localize to the same structures, pointing toward the heterogeneous nature of dendritic lysosomes. We found that the two most prominent splice isoforms of LAMP2, LAMP2A and LAMP2B, predominantly localize to the same membranes.

To gain further insight into the connection between synaptic signaling and lysosomal function, we focused on a key receptor for learning and memory processes, the GRIN/NMDAR (glutamate ionotropic receptor NMDA type) receptor. Live imaging experiments revealed that active lysosomes transiently visit the GRIN/NMDAR subunit GRIN2B/GluN2B. Following stimulation of GRIN/NMDAR, however, we observed a drastic decrease of lysosome motility, and lysosomal stalling results almost invariably in fusion with the plasma membrane.

We next asked about the molecular mechanism mediating stalling of LAMP2B-positive lysosomes at synaptic sites and we found a direct association between the membrane-associated guanylate kinases (MAGUKs) DLG3/SAP102 and LAMP2B. DLG3/SAP102 is a scaffold that clusters and anchors GRIN/NMDAR. When we interrupt this interaction with a cell-penetrating TAT peptide, GRIN/NMDAR-dependent lysosomal stalling and plasma membrane fusion are significantly reduced in neuronal cultures. A *DLG3* gene knockout in primary neurons has a similar effect.

**Figure 1.**
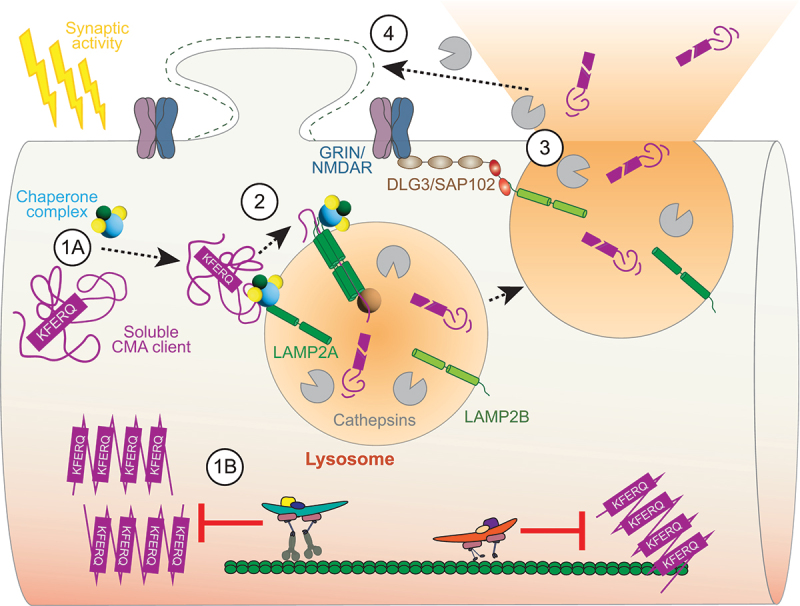
Activity-dependent lysosomal exocytosis of CMA clients. (1A) dendritic CMA is regulated by neuronal activity – the chaperone complex selectively targets CMA clients to the lysosomal lumen via (2) oligomerized CMA receptor LAMP2A. (1B) the impairment of this process may lead to protein aggregation resulting in disruption of intracellular trafficking, a scenario especially relevant for supersaturated, metastable proteins. (3) exocytic lysosomes bind via LAMP2B to the GRIN/NMDAR scaffold, DLG3/SAP102, and subsequently fuse with the plasma membrane releasing CMA clients and active proteases to the extracellular space. (4) the release of lysosomal proteases may contribute to the structural plasticity of spine synapses.

What is the functional role of activity-dependent dendritic lysosomal stalling and fusion? We and others observed that lysosomal fusion induces dendritic spine growth which is commonly observed following potentiation of spine synapses. Spine growth is probably mediated by cleavage of components of the extracellular matrix (ECM) due to the exocytotic release of lysosomal proteases. We next wondered what additional content might be released by exocytotic lysosomes. LAMP2A is a unique receptor for CMA, a selective form of autophagy whose substrates are soluble proteins harboring the KFERQ motif that is recognized by chaperones. Subsequently, intralysosomal and cytoplasmic chaperones orchestrate transient oligomerization of LAMP2A to facilitate the loading of the cargo directly into the lysosomal lumen. Using a CMA activator and chaperone inhibitor, we first determined the recruitment of a CMA sensor to the dendritic LAMP2A- and LAMP2B-positive lysosomes. Next, we demonstrated that dendritic CMA is downstream of GRIN/NMDAR activation and lysosomal fusion. Importantly, CMA clients often belong to the disease-relevant, metastable proteome, whose physiological concentration is close to solubility limits. Therefore, we next focused on the canonical CMA client TARDBP/TDP-43, whose aberrant function is associated with frontotemporal dementia. Super-resolution STED microscopy and live imaging experiments revealed that TARDBP indeed localizes to the LAMP2B-positive lysosomes. Finally, we could show that LAMP2B-DLG3/SAP102 binding of dendritic lysosomes mediates activity-dependent lysosomal exocytosis of two disease-relevant CMA clients: TARDBP and HTT, whose mutation results in Huntington disease (Figure 1).

While the activation of GRIN/NMDAR leads to lysosomal exocytosis, it is currently not clear whether these receptors localize to synaptic or extrasynaptic sites. Our data indicate that exocytosis mainly occurs in the dendritic shaft; however, it cannot be excluded that fusion also happens within excitatory shaft synapses. This is an important question because activation of extrasynaptic receptors usually occurs either following glutamate spillover in response to high-frequency stimulation or in disease states that are associated with a buildup of ambient glutamate. Thus, the findings might have implications for brain pathology. Under physiological conditions, lysosomal exocytosis in dendrites will likely serve in the local regulation of protein content in response to synaptic activity. It is highly interesting that heightened synaptic activity couples to lysosomal exocytosis of CMA clients belonging to the metastable subproteome that is implied in neurodegenerative diseases. In this regard, because supersaturation is a function of volume, thin dendritic processes are particularly vulnerable to aggregation of this subproteome. In fact, the dual function of lysosomal exocytosis may account for two seemingly distinct phenomena common for age-related neurodegenerative diseases – synaptic impairment and protein aggregation. The activation of glutamatergic receptors drives lysosomal fusion, whereas fusion then leads to the release of active proteases and an increase in dendritic spine volume. Moreover, it is likely that the underlying digestion of the extracellular matrix (ECM) will have further implications for synaptic plasticity. The ECM acts as a diffusion barrier for glutamate receptors, and its removal enables lateral diffusion from extrasynaptic to synaptic sites, a mechanism that previous work has implicated in an increase of synaptic strength. Collectively, our findings point to an intriguing scenario and a provocative hypothesis: Certain forms of synaptic plasticity may be an inevitable consequence of the need for local disposal of the metastable proteome.
